# Complications From Subdural Drains in Burr Hole Drainage of Chronic and Subacute Subdural Haematomas: A Single-Centre Experience

**DOI:** 10.7759/cureus.39068

**Published:** 2023-05-16

**Authors:** Sotirios Apostolakis, Konstantinos Vlachos

**Affiliations:** 1 Department of Neurosurgery, KAT General Hospital of Attica, Neo Faliro, GRC; 2 Department of Neurological Surgery, KAT General Hospital of Attica, Kifisia, GRC

**Keywords:** morbidity, subdural, hematoma, drain, complication

## Abstract

Introduction

The use of drains following the burr-hole evacuation of chronic (CSDH) and subacute subdural haematomas (SASDH) is a well-established practice offering a significant reduction in recurrence rates and improvement of survival. The purpose of this work is to investigate the complication rate of subdural drains following the burr-hole evacuation of CSDH and SASDH.

Methods

A retrospective review of the clinical records of all patients managed surgically for CSDH or SASDH was conducted. Patients over 18 years, that met the criteria for surgical evacuation, were included in this study. Patients admitted for CSDH or SASDH but managed either conservatively or with craniotomy were excluded from further analysis.

Results

A total of 97 cases, with a mean age at the time of diagnosis of 78.25 years were identified in which 122 drains were used. Three complications, two acute subdural haematomas, and one case of drain-associated seizures were identified, yielding an overall complication rate of 3%.

Conclusion

The use of intradural drains is associated with a small, yet not negligible, possibility of serious complications.

## Introduction

Subdural haematomas in their subacute (SASDH) or chronic (CSDH) phase are commonly encountered by neurosurgeons in the emergency setting and their burr-hole drainage is one of the first surgical skills a young neurosurgeon should master. Even though it is a relatively straightforward procedure, several alterations to the technique are employed. One of them regards the positioning of a subdural catheter to assist in the drainage of the residual haematoma.

The advantages of drain placement following the burr-hole evacuation of CSDH and SASDH have been well-documented in a number of studies [1-3]. These studies suggest that subdural drains significantly reduce recurrence and therefore reoperation rates, while at the same time, they have been associated with improved survival rates [4]. This was also demonstrated in several recent meta-analyses [5,6], rendering subdural drain placement a common practice among neurosurgeons.

Subdural drains are generally considered safe, with previous studies reporting a low incidence of complications associated with them, nevertheless, they fail to associate them purely with the drains per se [5,7]. Potential complications include but are not limited to, cutaneous and intracranial infections, acute bleeding, and parenchymal injury due to misplacement [7,8]. The purpose of this work is to evaluate the safety of subdural drains following the burr-hole evacuation of CSDH and SASDH in a tertiary clinical setting.

## Materials and methods

Participants

In the present study, a retrospective review of the clinical records of all patients admitted in the Department of Neurosurgery or the Intensive Care Unit (ICU) with the diagnosis of chronic or subacute subdural haematoma of either traumatic (ICD 10 code S06.5) or non-traumatic (ICD 10 code I62.0) aetiology from January 1, 2016 to December 31, 2018 was conducted.

A complete medical history was taken at the time of admission that included but was not limited to, any known medical conditions, medications, as well as any previous operations.

Patients over 18 years, with the diagnosis of either CSDH or SASDH, that met the criteria for surgical evacuation, as summarised in [9] and were treated with burr-hole drainage were included in this study. Patients admitted for CSDH or SASDH but managed either conservatively or with craniotomy were excluded from further analysis.

Whenever possible, the patient would be operated on the day of presentation at the Accident and Emergency Department by the attending neurosurgeon and the resident on call. Even though some minor differences exist among the practice of surgeons, the general procedure was fairly standard.

Procedure

Prior to skin incision, patients were administered a single dose of 1gr of IV cefoxitin, which was continued for three days postoperatively three times per day. In case of a known history of allergy to penicillin, clarithromycin 500mg b.i.d. was preferred.

In the operating theatre, patients would be placed in the supine position, with their heads on a horseshoe rest. Depending on the patient’s neurological status general endotracheal anaesthesia or local anaesthesia was administered, with the latter being reserved for patients in a coma (GCS<8).

**Figure 1:**
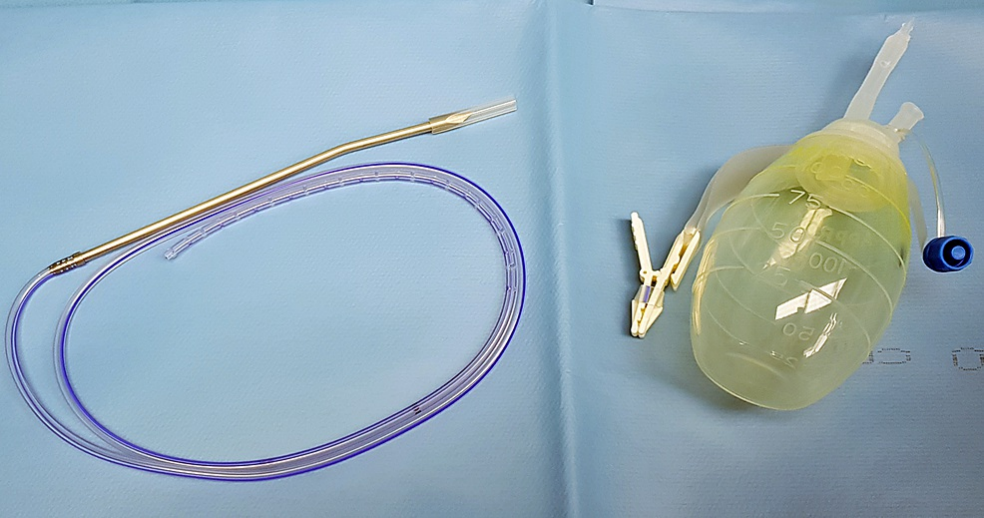
The standard Jackson-Pratt closed wound suction drainage system used in our practice.

Depending on the surgeon’s preferences, either a single enlarged temporal burrhole or two, one located frontally and one parietally, burr-holes over the maximum width of the haematoma were performed. Following meticulous haemostasis, the dura would be opened in a cruciate fashion and the haematoma would be drained. Thorough irrigation with warm normal saline of any residual haematoma would then be performed and the drain would be placed subdurally (Figure 1). In our institution, with the exception of a single case in which a 7mm wide catheter was selected, a Jackson-Pratt Closed Wound Suction Drainage System consisting of a 100cc bulb reservoir connected with a catheter with an external diameter of 3.3mm and an internal diameter of 1.5mm is typically used. As the negative pressure exerted is not fixed and depends on the distortion of the reservoir, with a maximum value of -97.9 cmH_2_O [10], only half of it was routinely squeezed leading to a suction pressure of about 41.3 cmH_2_O. Depending on the surgeon’s preference, some patients with unilateral haematomas were managed with two subdural drains, typically directed one frontally and one occipitally. The catheter would exit the skin about 2cm medially from the incision.

Whenever possible, patients were mobilised on the first postoperative day. In addition, on the day following the operation, an unenhanced brain CT scan would be obtained, and the subdural drain would be removed, with the skin integrity being restored with a single silk 2-0 suture. Re-evaluation one month following discharge in the Outpatients Department with a new brain CT scan would be suggested.

Data analysis

Clinical records of all patients were screened, and data were extracted individually by both researchers. Data of interest were age, gender, diagnosis, neurological status at the time of presentation as well as potential complications associated with the drain and recurrence of the haematoma.

Student’s T-test and chi-square were used to test for differences between groups for parametric and non-parametric variables respectively. The level of statistical significance was p < 0.05.

Statistical analysis was conducted using STATISTICA 10.0 (StatSoft 1984-2010) and MATLAB 2016 (The MathWorks, Inc., 2016). Figures were created using MATLAB 2016 (The MathWorks Inc., 2016) and Adobe Illustrator CS3 (Adobe Systems, 2007).

## Results

**Figure 2:**
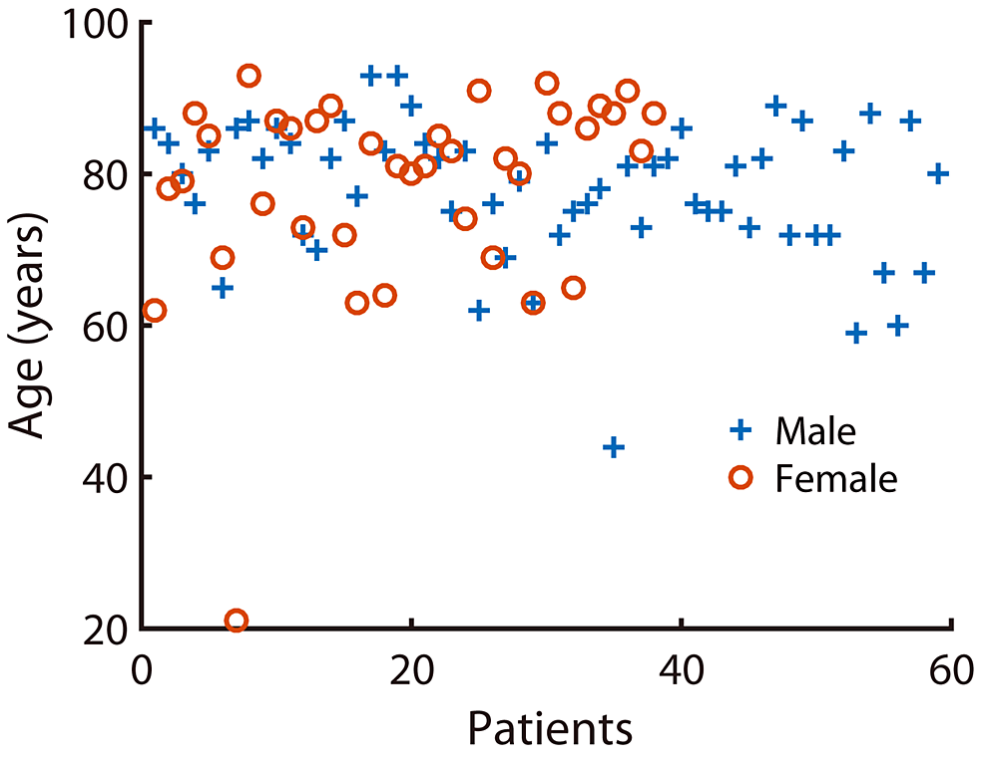
Age profiles of patients included in this study.

The records search yielded 97 cases with a total of 122 drainage systems being placed. The mean age at the time of diagnosis was 78.25 years, with a range of 21-93 years (Figure 2). Compared to an expected even distribution of cases, there were significantly more male patients than female patients (p<0.05). Age profiles of males (mean 77.9, SD 9.18) and females (mean 78.8, SD 13.2) did not differ significantly between the two genders (p=0.68). The demographic characteristics of the participants are presented in Table 1.

**Table 1:** Participants' demographics.

Demographics	
N	97
Gender (Male/Female)	59/38
Age in years Mean (SD)	78.25 (10.88)
Age in years Median (Range)	81 (21-93)
Unilateral haematoma	72
Bilateral haematoma	25
Total drainage systems placed	122

In total, we were able to identify three cases (2.5% per drain used) of major complications associated with intradural drain insertion. One case of focal seizures on the first postoperative day, following emptying of the drain and re-establishment of the negative pressure was recorded. We hypothesised that the catheter caused a small cortical contusion, which nevertheless was not evident in the CT scan that followed. The patient was put on levetiracetam 500mg twice daily and no further incidents were reported throughout the follow-up period.

In addition, two cases of subdural bleeding were documented, one intraoperative after placing the drain catheter and establishing the negative pressure and another one on the first postoperative day following removal of the drain. It is worth stressing the fact that the former patient was the single case in whom a 7mm catheter was used and the latter patient had two subdural drains. Both patients needed surgical evacuation of the acute haematomas and one of them finally succumbed to his complication, in spite of being otherwise in good health.

## Discussion

In the present study, we present a single-centre experience with the use of subdural drains following the burr-hole evacuation of CSDH and SASDH. An overall 3% rate of serious complications was found.

The complication rate was similar to other previously reported [3,11]. Rohde et al. report an overall complication rate of 20.5% but without explicitly reporting those associated with the drainage system [12]. The most frequent major complication was seizures, which were found in as many as 13.6% of all patients [12], yet the most common overall complication is local wound infection, which nonetheless, was not observed in any of our patients. There is largely consensus in the scientific community that drains placement is not associated with an increased risk for unfortunate events [2], something which was also found in a meta-analysis by Alcala-Cerra et al. [13]. Nevertheless, it appears that their nature and severity vary significantly. While in the non-drainage group complications are mostly dominated by superficial or intracranial infections, in the intervention group intra- or extra-axial haemorrhages as well as intraparenchymal placement of the drain are added to the list, which may have deleterious effects on the patient’s survival.

As drain insertion is typically a freehand and, mostly, blind technique with the exception possibly of endoscopic drainage, misplacement could be found in as many as 17% of patients [14] while another study found that 12.3% of them can be symptomatic [15]. In order to avoid such unfortunate events, Jensen et al. [16] suggest folding the tube before inserting it and letting it rest on the inner membrane of the haematoma once intracranially.

Surgical treatment of CSDH is typically an operation performed by residents under the guidance of the attending physician. Previous works have suggested that the involvement of residents in operations is associated with an increased risk for complications [17,18]; nevertheless, such operations are of paramount importance for their training in order to master basic neurosurgical skills.

An alternative to the subdural placement of the drain is the subperiosteal one. Opinions on their effectiveness and safety vary significantly, with some finding lower recurrence and complication rates [14] and others finding no difference at all [11,19]. A meta-analysis by Ding et al. [20] and another one by Greuter et al. [21] nevertheless, support the former opinion, suggesting that further research is still needed.

Even though the surgical treatment of CSDH and SASDH is one of the simplest operations in neurosurgery, there is no consensus over the optimal approach. Still, it appears that as far as recurrence rates are concerned, there is no superiority of two burr-holes over one and similarly of two over one drain [22], something which however has been challenged by some research groups [23]. Whether this practice has any effect on the complication rate, remains to be examined.

## Conclusions

The use of intradural drains is associated with a small, yet not negligible, possibility of serious complications, however it appears that the potential benefits significantly overweight the risks. Surgeons and patients alike should be well aware of the potential risks and benefits of an otherwise standard procedure.
